# Identification and assessment of factors that impact the demand for and supply of dental hygienists amidst an evolving workforce context: a scoping review

**DOI:** 10.1186/s12903-024-04392-6

**Published:** 2024-05-29

**Authors:** Mark J. Dobrow, Angela Valela, Eric Bruce, Keisha Simpson, Glenn Pettifer

**Affiliations:** 1 Institute of Health Policy, Management and Evaluation, Dalla Lana School of Public Health, University of Toronto, 155 College Street, Suite 425, Toronto, ON M5T 3M6 Canada; 2 Accessing Centre for Expertise, 155 College Street, Suite 425, Toronto, ON M5T 3M6 Canada; 3 College of Dental Hygienists of Ontario, 175 Bloor Street East, North Tower, Suite 601, Toronto, ON M4W 3R8 Canada

**Keywords:** Dental hygienist, Workforce, Supply, Demand, Scoping review

## Abstract

**Background:**

This study involved a scoping review to explore factors influencing dental hygienist demand and supply in high-income countries.

**Methods:**

A six-stage scoping review was conducted with separate search strategies tailored to four databases (MEDLINE, CINAHL, Google Scholar, and Google) plus a targeted scan of dental hygienist organization websites. This yielded 2,117 unique citations, leading to 148 articles included in the review.

**Results:**

Nearly half of the articles (47%) focused on the United States, with 11% on Canada. Most articles (91%) were in English, alongside 13 in Korean and one in French. Journal articles comprised 62% of the publications, followed by reports/working papers (11%) and websites (11%). Other types included conference abstracts, policy briefs, and presentation slides. Content-wise, 47% were original research, with analysis articles (14%), commentaries (11%), and reviews (8%) also present. The articles were coded into three main categories: workforce characteristics/projections, factor-specific analyses, and workforce opportunities. The articles on workforce characteristics covered demographic, geographic, and employment aspects of dental hygienists, along with projections for supply and demand using simulation modelling and geospatial analyses. Factor-specific articles investigated the (1) working environment, (2) policy/regulatory/training environment, (3) job/career satisfaction and related human resource issues, and (4) scope of practice. The third key category of articles highlighted opportunities for expanding the workforce through alternative models in different sectors/settings (e.g., public health, primary care, long-term care, hospitals, mobile outreach, and non-clinical roles including research, education and leadership) and for a range of vulnerable or underserved populations (e.g., geriatric and pediatric populations, persons with disabilities, those living in rural/remote areas, Indigenous peoples, and incarcerated people).

**Conclusions:**

This review provides a comprehensive documentation of the current state of the dental hygienist workforce, compiling factors affecting demand and supply, and highlighting opportunities for the dental hygienist workforce in Canada and other high-income countries. The findings offer a foundation for future research, highlighting the need for more focused and rigorous reviews and underscoring the necessity of high-quality studies to verify the effectiveness of various interventions and policies. This is crucial to address dental hygienist workforce challenges and ensure the sustainability and effectiveness of oral health care delivery.

**Supplementary Information:**

The online version contains supplementary material available at 10.1186/s12903-024-04392-6.

## Background

The dental hygiene profession, a key part of the oral health care field, has navigated through various challenges and transformations since it was established in the early 20th century [1]. Dental hygienists play a pivotal role in preventive oral health care, contributing significantly to population and public health.

Historically, the oral health care field has experienced considerable fluctuations in workforce dynamics, influenced by regulatory, educational, economic, and health-related factors. Some of the factors that have been noted include increases to dental hygiene education program capacity that lead to a higher number of qualified professionals entering the market; economic shifts that often influence health care funding, public spending on oral health, and employment opportunities for dental hygienists; and evolving scope-of-practice regulations and licensure requirements affecting where and how dental hygienists can practice [2–4]. These factors collectively contributed to a complex pre-pandemic workforce landscape, marked by regional disparities in the distribution of dental hygienists and varying employment opportunities [2, 5, 6].

The onset of the COVID-19 pandemic brought unprecedented challenges to the broader health care sector. The impact on the dental hygiene workforce was multifaceted, including health risks and safety concerns, economic effects, and clinical practice adaptations [7–9]. For example, dental hygienists faced high exposure risks due to the nature of their work, leading to increased safety concerns and work environment adjustments. The economic impact of the pandemic affected public spending on oral health and led to temporary closures or reduced operations in dental practices. The profession also adapted quickly to the need for enhanced safety protocols and tele-dentistry practices. Ultimately, there are indications that many dental hygienists left the profession during the pandemic, and post-pandemic, there is a growing perception of an undersupply of dental hygienists, attributed to factors such as workforce attrition, changes in work-life preferences, and ongoing safety concerns [10].

In light of this context characterizing the evolving pressures on the dental hygienist workforce, this study seeks to provide relevant insights for how high-income countries can move forward. With no existing or in-progress reviews on this topic registered with PROSPERO or Open Science Framework registries, this study aimed to conduct a scoping review to comprehensively explore the contemporary factors influencing dental hygienist demand and supply to contribute to a deeper understanding of the current state and future needs of this important oral health care profession.

## Methods

This study followed a six-stage scoping review adapted from the methodology first described by Arksey and O’Malley [11] and later refined with recommendations for additional methodological rigour by Levac et al. [12] and Daudt et al. [13]. Results of the scoping review are documented according to the Preferred Reporting Items for Systematic reviews and Meta-Analyses extension for Scoping Reviews (PRISMA ScR) [14] (Additional file 1).

### Stage 1. Identifying the review question

In the context of evolving workforce conditions for dental hygiene both pre and post COVID-19 pandemic, the main aim of the scoping review was to address the following review question: ‘What is known about the factors that impact the demand for or supply of dental hygienists in high-income countries’.

### Stage 2. Identifying relevant articles

A comprehensive search strategy involving four databases (Ovid-MEDLINE, EBSCO-Cumulative Index to Nursing and Allied Health Literature (CINAHL), Google Scholar, Google) was employed to identify relevant literature from both peer-reviewed and grey literature sources. MEDLINE and CINAHL databases were primarily utilized to access peer-reviewed research studies, offering a robust collection of academic articles across various dental hygiene and oral health care disciplines. Google Scholar served as a supplementary resource, providing access to a wide array of peer-reviewed journal articles, while also capturing a significant amount of grey literature, including reports published by government organizations, professional associations and regulatory agencies. Lastly, Google was used to broaden the search for grey literature, enabling identification of diverse sources such as working papers, reports, policy documents, conference abstracts, and unpublished studies. This multi-database approach allowed a thorough exploration of the available literature, while being mindful of scope and feasibility considerations.

Search strategies specific to each database were developed, using a mix of subject headings, keywords, and Boolean operators (where applicable). This resulted in similar search strategies for MEDLINE and CINAHL, and five distinct search strategies for both Google Scholar and Google (see Additional file 2 for the search strategies used for each of the four databases). Limits were not imposed for language or publication type and the eligible time period was set to go back no more than 10 years (i.e., articles published in 2013 or later) to focus on current factors that impact demand or supply. All searches were conducted in April and May 2023.

### Stage 3. Article selection

All search results were exported to and managed in an Excel database. Results from the MEDLINE and CINAHL searches were combined, and duplicates removed. Google Scholar and Google search results were also compared, and duplicates removed. Study selection involved screening of titles and abstracts to identify relevant articles that were then subjected to full-text assessment to determine the final set of articles to be included in the review.

Eligibility criteria to guide screening were developed by identifying a representative sample of 10 articles from preliminary searches and discussing their relevance with the research team. Based on the feedback, the following eligibility criteria were established:

- Articles must focus on dental hygienists (either exclusively or alongside other related professions).
- Articles must focus on factors impacting on demand for and/or supply of dental hygienists (e.g., accessibility, availability, capacity, workforce).
- Articles should focus on high-income jurisdictions where dental hygienists have a similar scope of practice as in Ontario, Canada.
- Articles must be published in the last 10 years (2013 to present).
- No article type restrictions.
- No article language restrictions.

For the MEDLINE and CINAHL search results, all identified titles/abstracts were screened for relevance based on the above eligibility criteria. All search results from the five Google search strategies (automatically excluding the Google omitted results) were screened based on titles, available excerpts and clicking on one weblink maximum. For the Google Scholar search, the first 100 results (where available) for each of the five search strategies were screened based on titles, available excerpts and clicking on one weblink maximum, with screening of additional results conducted in batches of 20 based on ongoing saturation assessment. If no new documents were identified in the subsequent batch, screening was stopped screening. All screening was conducted by one reviewer (AV) with a second reviewer (MJD) screening a subset of titles/abstracts from all four databases to calibrate the interpretation of the eligibility criteria. Any disagreements were discussed and resolved through consensus. Where necessary, DEEPL Translate or Google Translate was used to translate non-English titles/abstracts into English to facilitate screening.

For all records screened as potentially relevant, attempts were made to retrieve full-text versions for further assessment. DEEPL Translate was used to translate full-text articles originally published in a non-English language into English to facilitate the full-text assessment. All full-text articles were assessed by one reviewer (AV) with a subset reviewed by a second reviewer (MJD) for calibration purposes. Any disagreements were discussed and resolved through consensus. Full-text assessment was based on the same eligibility criteria used for title/abstract screening.

### Stage 4. Data extraction

A data extraction form was developed in Excel that included the following fields: citation information (including uniform resource locator – URL), publication type, article type, jurisdiction, sub-jurisdiction, methodological approach, target professions and/or populations, summary findings, summary conclusions, and future research/policy gaps. One reviewer (AV) conducted an initial extraction of data from the included articles, with the second reviewer (MJD) reviewing and updating the data extraction for all included articles.

### Stage 5. Synthesis

Once data extraction was complete, the extracted data were analyzed and synthesized through multiple lenses. This included documenting the distribution of identified articles by year, jurisdiction/sub-jurisdiction, publication type, article type, and publication language. One reviewer (MJD) thematically coded each included article based primarily on the summary findings and/or summary conclusions, with coding categories iteratively refined and synthesized to identify articles with similar focus. The research team reviewed and provided feedback on the thematic coding categories.

Given the intention to capture a mix of article types (i.e., the search was not restricted to original research or review articles), a formal quality appraisal of the included articles was not conducted. However, the research methodologies employed for the original research, analysis, and review articles were documented.

### Stage 6. Consultation

As the research team included several senior members of the Ontario College of Dental Hygienists with established networks and relationships with Canadian and international dental hygienist stakeholder organizations and leaders, their knowledge and perspectives provided an additional lens on the scoping review findings. The team’s networks were further leveraged to identify a selection of comparable Canadian and international dental hygienist organizations (e.g., professional associations and regulators) to include in a targeted scan of their websites to identify additional records. Table 1 provides the final list of targeted organizations for the scan.

**Table 1:** Jurisdictions/organizations targeted for scan

#	Jurisdiction	Target Organization	Website
1	Australia	Dental Board, Australian Health Practitioner Regulation Agency	https://www.dentalboard.gov.au
2	Australia	Dental Hygienist Association of Australia	https://dhaa.info
3	Canada	Canadian Dental Association	https://www.cda-adc.ca/en/index.asp
4	Canada	Canadian Institute for Health Information	https://www.cihi.ca/en
5	Canada	Federation of Dental Hygiene Regulators of Canada	https://www.fdhrc.ca
6	Canada	Ontario Dental Hygienists Association	https://odha.on.ca
7	Canada	The Canadian Dental Hygienists Association	https://www.cdha.ca
8	Canada	Ontario Dental Association	https://www.oda.ca
9	Canada	Ontario Ministry of Health	https://www.health.gov.on.ca/en/
10	Canada	Fédération des Hygienists Dentaires du Québec	https://fhdq.org
11	Canada	Ordre des Hygiéists Dentaires du Québec	https://ohdq.com
12	Japan	Japan Dental Hygienists’ Association	https://www.jdha.or.jp/en/
13	Korea	Korean Dental Hygienists Association	https://eng.kdha.or.kr
14	Switzerland	Swiss Dental Hygienists	https://dentalhygienists.swiss/home
15	United Kingdom	British Dental Hygienists and Dental Therapists Association	https://www.bsdht.org.uk
16	United States	American Academy of Dental Hygiene	https://www.aadh.org
17	United States	American Dental Hygienists Association	https://www.adha.org
18	United States	American Dental Political Action Committee	https://givetoadpac.ada.org
19	International	International Federation of Dental Hygienists	https://ifdh.org
20	International	International Symposium on Dental Hygiene 2022	https://isdh2022.com/

To capture publicly available information on these organizations’ websites, a two-pronged approach was taken. First, each website’s available search functionality was used to search for five principal keywords (i.e., workforce, supply/demand, capacity, access, availability); then, a manual scan of each website was conducted to identify relevant documents. Google Translate was used to translate non-English language websites to English to facilitate the targeted scan. Any identified articles or documents were integrated into the review and followed the methodology outlined above. All targeted websites were scanned between April and June 2023.

## Results

**Fig. 1:**
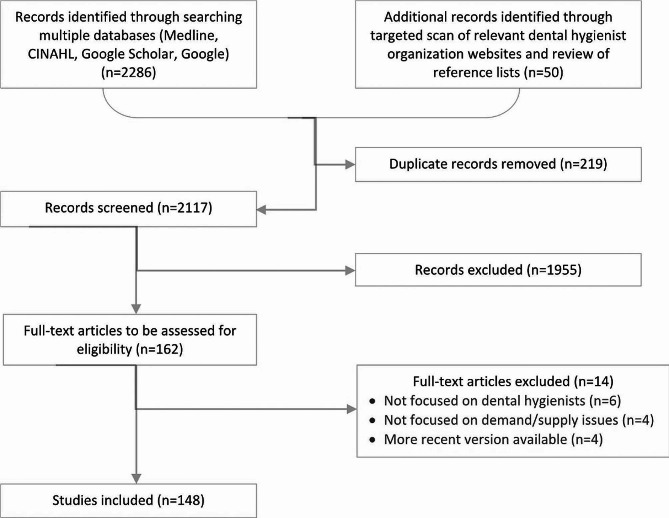
PRISMA-ScR flow diagram of scoping review results

The study identified 2117 unique records, of which 162 were flagged for full-text assessment after title/abstract screening. After full-text assessment, 148 articles/documents were included in the final review. Table 2; Fig. 1 provide an overview of the scoping review results at key stages of screening and review, while Additional file 3 provides a complete list of the 148 articles/documents including in the final review.

**Table 2:** Search results

Database	Search Results	Titles/Abstracts Screened (after Deduplication)	Articles/Documents Recommended/ Available for Full-Text Review	Articles/Documents Included in Final Review
Medline	316	686	50	47
CINAHL	543			
Google Scholar	580	569	50	45
Google	847	812	33	27
Targeted Scan	50	50	29	29
**Total**	**2336**	**2117**	**162**	**148**

Key characteristics of the 148 articles were documented (Table 3). The published articles were distributed relatively evenly over the last 10 years, with between 9 and 14 articles identified each year from 2013 to 2023, with exceptions being 2020 (17 articles) and 2022 (25 articles). The publication language was English for over 90% of the articles reviewed. Fourteen non-English language articles (13 Korean, one French), which each included an English language title/abstract, were included, and DEEPL Translate was used to generate a full-text English language version of these articles for the review.

**Table 3:** Characteristics of included articles

Publication Year		Jurisdiction of Focus		Publication Type		Article Type	
2013	9 (6%)	USA	69 (47%)	Journal articles	92 (62%)	Original research	69 (47%)
2014	14 (9%)	Canada	16 (11%)	Reports/working papers	16 (11%)	Analysis	21 (14%)
2015	11 (7%)	Korea	16 (11%)	Websites	16 (11%)	Commentaries	17 (11%)
2016	13 (9%)	UK	11 (7%)	Conference abstracts	9 (6%)	Reviews	12 (8%)
2017	11 (7%)	Australia	9 (6%)	Policy briefs	9 (6%)	Country profiles	11 (7%)
2018	13 (9%)	Japan	8 (5%)	Presentation slides	4 (3%)	Conference abstracts	9 (6%)
2019	13 (9%)	Sweden	3 (2%)	Other	2 (1%)	Policy guidance/information	3 (2%)
2020	17 (11%)	Finland	2 (1%)	**Total**	**143 (100%)**	Blogs	2 (1%)
2021	13 (9%)	Germany	2 (1%)			Other	4 (3%)
2022	25 (17%)	Hong Kong	2 (1%)				
2023	9 (6%)	Spain	2 (1%)	**Publication Language**			
		Switzerland	2 (1%)	English	134 (91%)		
		Ireland	1 (1%)	Korean	13 (9%)		
		International	5 (3%)	French	1 (1%)		
**Total**	**148 (100%)**	**Total**	**148 (100%)**	**Total**	**148 (100%)**	**Total**	**148 (100%)**

The intent was to focus on high-income jurisdictions with similar scope of practice for dental hygienists as Ontario, Canada. While 16 (11%) of the articles focused on Canadian jurisdictions, almost half of the included articles focused on the United States (USA) (47%), followed by Korea (11%), United Kingdom (UK) (7%), Australia (6%) and Japan (5%) (Table 3). Seven other countries were the focus of three or fewer articles, while an additional five articles had an international or multi-jurisdictional focus. Most of these jurisdictions have active dental hygienist professions and the review includes International Federation of Dental Hygienists country profiles for each of the represented countries where updated information on dental hygiene profession was available (exceptions: Hong Kong, Ireland).

Of the 148 articles included in the review, the most common publication type was journal articles (62%), followed by reports/working papers (11%), and websites (including blogs, policy information and country profiles) (11%) (Table 3). Other publication types included conference abstracts, policy briefs, and presentation slides. When examining the types of articles, almost half were original research (47%), followed by analysis articles (14%), commentaries (11%), and reviews (8%) (Table 3).

As noted in the summary of the methodological approach, there was no restriction on publication type or article type to allow for a broad range of contextual factors and perspectives to be considered. Further, a formal quality appraisal of included articles was not conducted. However, the research methodologies employed for the 102 original research, analysis, and review article types included in the review were documented (Table 4). For the 69 articles categorized as original research, the majority (58 studies) employed quantitative methods, including surveys, geospatial analyses, administrative data analyses, quasi-experimental studies, and electronic medical record reviews. Another 10 of the original research articles were based on qualitative research methods (e.g., interviews, focus groups, document review), while one other original research article was based on a mixed methods (qualitative/quantitative) approach. For the 21 articles categorized as analysis articles, the methodology was identified for 18. This included six articles describing workforce modelling methods (e.g., simulation modelling), several based on secondary analyses of existing data sets (four based on analysis of multiple data sources, three based on survey data from licensure renewal processes, two based on analysis of registrant or administrative data sources), two document reviews (e.g., state-by-state direct access legislation for dental hygienists), and one strategic review. The three remaining analysis articles drew on mixed data sources but did not specify any specific methods. Finally, of the 12 review articles, 10 were published in journal articles, one in a report, and one in a presentation slide. Only three of the review articles noted any specific review methodology (one systematic review, one scoping review, one narrative review).

**Table 4:** Methodological approach of original research, analysis and review articles

Original Research Articles		Analysis Articles		Review Articles	
Quantitative-survey	50 (72%)	Quantitative-modelling/workforce projections	6 (29%)	Narrative review	1 (8%)
Quantitative-geospatial analysis	3 (4%)	Quantitative-analysis of multiple data sources	4 (19%)	Scoping review	1 (8%)
Quantitative-administrative data analysis	2 (3%)	Quantitative-analysis of survey data from license renewal process	3 (14%)	Systematic review	1 (8%)
Quantitative-quasi-experimental studies	2 (3%)	Quantitative-analysis of registrant/ administrative data	2 (10%)	Methodology not specified	9 (75%)
Quantitative-electronic medical record review	1 (1%)	Document review	2 (10%)		
Qualitative-interviews	7 (10%)	Strategic review	1 (5%)		
Qualitative-focus groups	2 (3%)	Methodology not specified	3 (14%)		
Qualitative-document review	1 (1%)				
Mixed methods research	1 (1%)				
**Total**	**69 (100%)**	**Total**	**21 (100%)**	**Total**	**12 (100%)**

Thematic coding of the 148 included articles resulted in three primary categories, including (1) workforce characteristics or projections (27 articles), (2) factors impacting on the supply of or demand for dental hygienists (100 articles), and (3) opportunities to impact supply or demand for dental hygienists (21 articles).

## Discussion

The review identified 148 articles, which given the flexible eligibility criteria, included a wide range of publication and article types. Although the focus was on high-income countries where dental hygienists had a similar scope of practice to Ontario, Canada, there was still good diversity with 13 countries represented, including 85 articles (57%) from North America, 35 (24%) from Asia-Pacific, 23 (16%) from Europe/UK, and 5 (3%) with an international focus. Although over 91% of articles were published in English, it is important to note that 13 included articles were published in Korean. Korea has an active dental hygienist profession and associated academic sector, with several dedicated academic journals indexed by major scholarly databases publishing on dental hygiene in Korean. As the interest was in assessing factors impacting demand for and supply of dental hygienists that extended both before and after the COVID-19 pandemic, it is also notable that there was a reasonable balance between the 84 (57%) articles published before 2020, and the 64 (43%) articles published from 2020 to 2023. This latter time period is an imperfect proxy for the pandemic/post-pandemic period given that that some articles published in 2020 may have been prepared pre-pandemic. It is also worth noting that while the recent uptick in published articles might be explained by broader shifts in publication rates during the COVID-19 pandemic, the search results indicate that dental hygienist demand/supply issues have received limited but consistent attention over the last decade.

Beyond the descriptive results of the scoping review, thematic coding of the included articles was key to synthesizing the findings. The key themes captured articles that focused on core workforce characteristics and projections, a wide range of factors impacting on demand and supply, and opportunities for the dental hygienist workforce. The following sections summarize the key findings by thematic category.

### Dental hygienist workforce characteristics/projections

Several articles were identified that described basic demographic (e.g., age, gender), geographic (e.g., practice location and distribution), and employment (e.g., part-time vs. full-time) characteristics of the dental hygienist workforce in different jurisdictions. This included Australia [15], Canada [16–18], New Zealand [19], Korea [20], Sweden [19], the UK [3], and the USA (including specific states such as Florida [21], Indiana [22], Iowa [23], Massachusetts [24], and Minnesota [25]). Several other articles went a step further, using different methodological approaches to provide projections for both the demand for and supply of dental hygienists and to assess the resultant gaps (i.e., gap analysis). This included simulation modelling for the USA [26–28], geospatial analyses for Australia [29] and Germany [30], and two comprehensive workforce assessments for Hong Kong [31, 32] and one for the UK [5].

Most workforce projections prior to the COVID-19 pandemic indicated an oversupply of dental hygienists, however, this was not universal. For example, one American analysis in 2015 suggested an oversupply of dental hygienists in all but five states (Mississippi, Montana, North Dakota, South Dakota, West Virginia) [26] while two analyses of Hong Kong’s dental hygienist workforce in 2014 and 2017 projected a shift from an oversupply to undersupply of dental hygienists [31, 32], and another analysis of the UK’s oral health care workforce in 2014 projected a substantive undersupply of dental hygienists [5]. Assessments conducted at the height of the COVID-19 pandemic or thereafter, suggested important shifts to the dental hygienist workforce with undersupply a major concern [10, 33]. Two geospatial analyses, both conducted pre-COVID-19 pandemic, suggested variable distributions with respect to population and/or socio-economic distributions [29, 30]. While the articles reviewed describe key characteristics and workforce projections from many high-income jurisdictions, collectively, they do not provide a clear general pattern for shifting dental hygienist demand or supply over the last decade.

### Factors impacting on dental hygienist demand/supply

Beyond the basic assessment of workforce characteristics and projections, the majority of articles identified examined various factors that impact the demand for or supply of dental hygienists. The key types of factor identified can be categorized as (1) working environment factors, (2) policy/regulatory/training environment factors, (3) job/career satisfaction factors and related human resource issues, and (4) scope of practice factors.

#### Working environment factors

Seven articles focused on the dental hygienist working environment, with two Canadian studies providing evidence of high rates of workplace bullying, harassment, abuse and/or violence by employers, colleagues or clients [34, 35]. Three articles addressed the impacts of the COVID-19 pandemic on the dental hygienist workforce in the USA [8, 36] and Spain [9], while two other articles addressed mental health challenges for dental hygienists in Sweden [37] and the UK [38]. Each of these articles outline important working environment factors that impact workforce supply.

#### Policy/regulatory/training environment factors

The review included several articles that captured policy, regulatory and/or training environment factors. Three articles described USA-state level regulatory environments, two which focused on licensure requirements [39] or license portability [40] and a third that presented an examination of the relationship between state-specific workforce policies and underserved individuals’ access to oral health care services [41]. The review also captured 11 country profiles that outline jurisdiction-specific requirements to work as a dental hygienist. Note that not all country profiles were current and any that were last updated before 2013 were excluded [42–52]. Several articles covered dental hygienist training program changes, including an article on survey results for motivations for pursing a baccalaureate degree [53], four articles commenting on the education needs of the profession [54–57], and four articles on the impacts of higher education requirements [4, 58–60]. A Korean study described the development of a ‘job performance assessment tool’ that aimed to align evolving performance needs with training curricula [61].

#### Job/career satisfaction factors / human resource issues

The review identified many articles that targeted various aspects of dental hygienist job/career satisfaction or related human resource issues in different jurisdictions. These factors all relate directly to workforce supply. For example, five Korean articles and three Japanese articles focused on aspects of dental hygienist mobility, turnover and return-to-work [62–69]. Five articles also addressed dental hygienist burnout, including two from the USA [70, 71], and one from Canada [72], Ireland [73], and Korea [74]. A survey conducted in Iowa, USA assessed factors contributing to multiple job-holding scenarios for dental hygienists [75]. Another article from the USA reported on a survey of dentists regarding their challenges in recruiting dental hygienists post-COVID-19 pandemic. Finally, another series of articles focused on various enablers or components of dental hygienist job/career satisfaction. This included two from Korea [76, 77], and one each from Australia [78], Japan [79] and the USA [80].

#### Scope of practice factors

Scope of practice was a key focus of a number of the articles reviewed. This included a wide mix of article types (e.g., original research, secondary analyses, reviews, commentaries) and research methods employed (e.g., broad range of qualitative and quantitative methods). The majority of articles on scope of practice were from the USA, but six other countries were represented (Australia, Canada, Finland, Japan, Switzerland, UK). These scope of practice articles primarily focused on (1) expansion of the dental hygienist scope of practice, (2) direct access / independent practice, and (3) misunderstanding of the dental hygienist role and scope of practice.

Articles on the expansion of the dental hygienist scope of practice included a survey of extended practice dental hygienists in Oregon, USA that identified key barriers to optimizing the extended practice permit available in that state [81]. That study found that lack of business knowledge, lack of experience, insurance reimbursement challenges and start-up costs were all barriers that limited the utilization of the expanded scope of practice that could be addressed through adaptation to training curricula and internship opportunities. Another article noted legislative efforts in Arizona, USA to facilitate delivery and billing of dental hygiene services in medical environments (e.g., hospitals, long-term care facilities) [82]. Several other articles addressed the impact on outcomes when the dental hygienist scope of practice was extended or expanded. A geospatial analysis drawing primarily on administrative data in the 13 state Appalachia region of the USA found that areas with low availability of dentists and dental hygienists had significantly lower dental service provision [83]. Several articles reported on analyses that linked broader scope of practice for dental hygienists to improved oral health outcomes among US states [84–86], while another American study noted that expanded roles for dental hygienists could have positive impacts on specific populations, such as low-income children in the state of Kansas [87]. Finally, an Australian survey of dental hygienists, dental therapists and oral health therapists compared the extent to which each profession was able to reach its full scope of practice [88]. The results suggested that in the Australian context, only the oral health therapists reached their full scope, but the study also provided important insights for optimizing scope of practice for jurisdictions, like Ontario, that that do not have active dental therapist or oral health therapist professions.

There were a number of articles that focused on direct access or independent practice for dental hygienists. There were a few historical summaries and/or documentation of legislative and policy achievements in the USA and UK on the introduction of direct access for dental hygienists [89–92], with one highlighting the variation across US states on direct access provisions [90]. A UK-based study in Wales assessed the range of oral health needs of care home residents that could be wholly provided by dental hygienists or dental therapists without oversight from a dentist [93]. The study indicated that 22% of care home resident oral health treatment needs could be provided by a dental hygienist without dentist supervision, and that increased to 43% for dental hygienists with special care experience, suggesting important potential impacts if direct access policies are optimized. A few other articles focused on the levels of professional autonomy and decision-making capacity required to optimize direct access to dental hygienist care. One US-based survey of eight states indicated that age, education level, and gender may affect a dental hygienist’s level of autonomy, but overall, registered dental hygienists are prepared to work independently [94]. Another US-based study examined different levels of autonomy from direct supervision to full independence and found that the most consistent increases in utilization of independent practice was associated with preventive care services, rather than more intensive treatments [95]. A Canadian study surveyed dental hygienists and suggested that decision-making capacity for independent practice may have some correlation with length of educational training [96]. Related, two commentaries, one from the US [97] and one from the UK [98], both indicated that advancing direct access or independent practice policies requires dental hygienist training programs to adapt educational curricula and incorporate more opportunities for practical experience and interprofessional education.

In light of expansion of the scope of practice for dental hygienists, there was also a collection of articles from four different countries that highlighted misunderstandings of the dental hygienist role and scope of practice as part of a broader dental team. A commentary on the American context noted the importance of re-positioning the role of dental hygienists ‘beyond cleaning teeth’ [99], while a UK-focused commentary highlighted the lack of recognition of dental hygienist skills and the associated negative impacts on the profession and oral health outcomes [100]. Two original research articles also emphasized the misunderstanding of the dental hygienist role. A Finnish qualitative study involved interviews with dental hygienist educators and noted the mismatch between the role of dental hygienists in dental teams and their evolving scope of practice [101]. A Japanese survey noted a lack of clarity on dental team members’ roles/scope of practice [102].

### Opportunities for the dental hygiene workforce

Another important thematic category captured opportunities to expand supply of the dental hygienist workforce through alternative workforce models involving different sectors/settings and populations. One American author summarized the evolving opportunities for dental hygienists with the following editorial extract:

*“As our health care system continues to evolve, I feel confident there will be increasing opportunities for dental hygienists to contribute to the triple aim of improving patient experiences, improved population health, and reduced costs of care. Dental hygienists can contribute to achieving the triple aim through expanded scope of practice especially for vulnerable populations; integration into primary care and other alternative settings such as long-term care facilities, hospitals, community-based programs, and home residences; and through leadership roles at the local, state, and national levels.”* [103].

Reference to alternative models was also evident in several other USA-based articles [6, 104–106], with focus ranging from analyses of alternative workforce models [104] to the requirements to set up and operationalize them [105, 107, 108] to the importance of collaborative practice [97, 109].

There were many calls for the dental hygienist profession to be prepared for new opportunities, particularly related to different sectors/settings and populations. Reviewed articles acknowledged opportunities in public health [110, 111], primary care [112], long-term care [113], hospitals [114], and mobile outreach [115]. Two articles also highlighted non-clinical roles including research, education and leadership [2, 103]. Common themes across all these articles were the need to learn new skills and work with interprofessional teams.

The reviewed articles also identified specific populations that could represent greater potential demand for dental hygienist services, including a range of vulnerable or underserved populations, such as geriatric [93, 113, 116–120] and pediatric [87, 116, 121–123] populations, persons with disabilities [124], those living in rural/remote areas [107, 115, 125], Indigenous peoples [115, 125], and incarcerated people [126].

Lastly, a recent submission from the Canadian Dental Hygienists Association to the Canadian House of Commons Standing Committee on Health anticipated the important role dental hygienists can play as part of the federal government’s new investment into a federal dental program [127].

### Limitations

Several methodological limitations should be considered. First, to encompass a wide range of issues affecting dental hygienist demand and supply, the review permitted various article types, including non-research articles. This approach, however, meant that the methodological quality of all included articles could not be assessed, particularly non-research articles like commentaries. However, the methodologies of the 102 original research, analysis, and review articles were all documented. Second, the focus on high-income countries, while relevant to the overarching objectives, limits the applicability of the findings for lower- and middle-income countries. Third, by restricting the review to the past 10 years, the aim was to capture current challenges and solutions, but this may have omitted important studies published before 2013. Lastly, the review process, primarily conducted by one researcher, could potentially introduce biases or inconsistencies. To mitigate this, a second reviewer screened and assessed a subset of records, to calibrate the application of the review eligibility criteria.

## Conclusions

This scoping review systematically explored the factors impacting the demand for and supply of dental hygienists, focusing primarily on high-income countries. The review aimed to identify key factors impacting this workforce, understand regional variations, and provide insights into future trends. The review encompassed 148 articles, with 102 employing a range of research methodologies representing 13 high-income jurisdictions but was predominated by surveys from the United States. Ultimately, the review provided a comprehensive documentation of the current state of the dental hygienist workforce, compiling factors affecting demand and supply, and highlighting opportunities for the dental hygienist workforce in Canada and other high-income countries. The findings offer a foundation for future research, highlighting the need for more focused and rigorous reviews and underscoring the necessity of high-quality studies to verify the effectiveness of various interventions and policies. This is crucial to address dental hygienist workforce challenges and ensure the sustainability and effectiveness of oral health care delivery.

### Electronic supplementary material

Below is the link to the electronic supplementary material.

Supplementary Material 1

## Data Availability

All data generated or analysed during this study are included in this published article (and its Additional File 2, Additional File 3).

## Abbreviations

CINAHL: Cumulative Index to Nursing and Allied Health Literature
COVID-19: Coronavirus Disease 2019
PRISMA ScR: Preferred Reporting Items for Systematic reviews and Meta-Analyses extension for Scoping Reviews
UK: United Kingdom
URL: Uniform Resource Locator
USA: United States of America
