# Supplementary material for: Identification and assessment of factors that impact the demand for and supply of dental hygienists amidst an evolving workforce context: a scoping review
Source: BMC Oral Health. 2024 May 29;24:631. doi: 10.1186/s12903-024-04392-6 (PMC11137971; doi:10.1186/s12903-024-04392-6)
Supplement: Supplementary file 1 — Supplementary Material 1 [file 12903_2024_4392_MOESM1_ESM.docx]

**BMC Oral Health**

**Title Page**

**Supplementary Files**

**Manuscript Title:**

Identification and assessment of factors that impact on the demand for and supply of dental hygienists amidst an evolving workforce context: a scoping review

**Authors:**

Mark J. Dobrow^1,2^*

Angela Valela^2^

Eric Bruce^3^

Keisha Simpson^3^

Glenn Pettifer^3^

^1^Institute of Health Policy, Management and Evaluation

Dalla Lana School of Public Health

University of Toronto

155 College Street, Suite 425

Toronto, ON M5T 3M6

^2^Accessing Centre for Expertise

155 College Street, Suite 425

Toronto, ON M5T 3M6

^3^College of Dental Hygienists of Ontario

175 Bloor Street East
North Tower, Suite 601
Toronto, ON M4W 3R8

*Corresponding author

**Keywords**

Dental hygienist; workforce; supply; demand; scoping review

# Supplementary Information

## Additional File 1: Preferred Reporting Items for Systematic reviews and Meta-Analyses extension for Scoping Reviews (PRISMA ScR)

| **SECTION** | **ITEM** | **PRISMA-ScR CHECKLIST ITEM** | **REPORTED ON PAGE #** |
| --- | --- | --- | --- |
| **TITLE** |  |  |  |
| Title | 1 | Identify the report as a scoping review. | Title page (Page 1) |
| **ABSTRACT** |  |  |  |
| Structured summary | 2 | Provide a structured summary that includes (as applicable): background, objectives, eligibility criteria, sources of evidence, charting methods, results, and conclusions that relate to the review questions and objectives. | Abstract (Page 2) |
| **INTRODUCTION** |  |  |  |
| Rationale | 3 | Describe the rationale for the review in the context of what is already known. Explain why the review questions/objectives lend themselves to a scoping review approach. | Background (Page 3) |
| Objectives | 4 | Provide an explicit statement of the questions and objectives being addressed with reference to their key elements (e.g., population or participants, concepts, and context) or other relevant key elements used to conceptualize the review questions and/or objectives. | Methods/Stage 1 (Page 4) |
| **METHODS** |  |  |  |
| Protocol and registration | 5 | Indicate whether a review protocol exists; state if and where it can be accessed (e.g., a Web address); and if available, provide registration information, including the registration number. | n/a |
| Eligibility criteria | 6 | Specify characteristics of the sources of evidence used as eligibility criteria (e.g., years considered, language, and publication status), and provide a rationale. | Methods/Stage 3 (Pages 4-5) |
| Information sources* | 7 | Describe all information sources in the search (e.g., databases with dates of coverage and contact with authors to identify additional sources), as well as the date the most recent search was executed. | Methods/Stage 2 (Page 4) |
| Search | 8 | Present the full electronic search strategy for at least 1 database, including any limits used, such that it could be repeated. | Additional File 2 – all search strategies provided |
| Selection of sources of evidence† | 9 | State the process for selecting sources of evidence (i.e., screening and eligibility) included in the scoping review. | Methods/Stage 3 (Pages 4-5) |
| Data charting process‡ | 10 | Describe the methods of charting data from the included sources of evidence (e.g., calibrated forms or forms that have been tested by the team before their use, and whether data charting was done independently or in duplicate) and any processes for obtaining and confirming data from investigators. | Methods/Stage 4 (Page 5) |
| Data items | 11 | List and define all variables for which data were sought and any assumptions and simplifications made. | Methods/Stage 4 (Page 5) |
| Critical appraisal of individual sources of evidence§ | 12 | If done, provide a rationale for conducting a critical appraisal of included sources of evidence; describe the methods used and how this information was used in any data synthesis (if appropriate). | Methods/Stage 5 (Page 5); Results (Page 8) |
| Synthesis of results | 13 | Describe the methods of handling and summarizing the data that were charted. | Methods/Stage 5 (Page 5) |
| **RESULTS** |  |  |  |
| Selection of sources of evidence | 14 | Give numbers of sources of evidence screened, assessed for eligibility, and included in the review, with reasons for exclusions at each stage, ideally using a flow diagram. | Results; Table 2; Figure 1 (Pages 6-7) |
| Characteristics of sources of evidence | 15 | For each source of evidence, present characteristics for which data were charted and provide the citations. | Results; Table 3; Table 4 (Pages 7-8) |
| Critical appraisal within sources of evidence | 16 | If done, present data on critical appraisal of included sources of evidence (see item 12). | Results (Page 8) |
| Results of individual sources of evidence | 17 | For each included source of evidence, present the relevant data that were charted that relate to the review questions and objectives. | Results (Pages 7-9) |
| Synthesis of results | 18 | Summarize and/or present the charting results as they relate to the review questions and objectives. | Discussion (Pages 9-12) |
| **DISCUSSION** |  |  |  |
| Summary of evidence | 19 | Summarize the main results (including an overview of concepts, themes, and types of evidence available), link to the review questions and objectives, and consider the relevance to key groups. | Conclusions (Page 13) |
| Limitations | 20 | Discuss the limitations of the scoping review process. | Discussion/Limitations (Page 12) |
| Conclusions | 21 | Provide a general interpretation of the results with respect to the review questions and objectives, as well as potential implications and/or next steps. | Conclusion (Page 13) |
| **FUNDING** |  |  |  |
| Funding | 22 | Describe sources of funding for the included sources of evidence, as well as sources of funding for the scoping review. Describe the role of the funders of the scoping review. | Declarations/Funding (Page 21) |

JBI = Joanna Briggs Institute; PRISMA-ScR = Preferred Reporting Items for Systematic reviews and Meta-Analyses extension for Scoping Reviews.

* Where *sources of evidence* (see second footnote) are compiled from, such as bibliographic databases, social media platforms, and Web sites.

† A more inclusive/heterogeneous term used to account for the different types of evidence or data sources (e.g., quantitative and/or qualitative research, expert opinion, and policy documents) that may be eligible in a scoping review as opposed to only studies. This is not to be confused with *information sources* (see first footnote).

‡ The frameworks by Arksey and O’Malley (6) and Levac and colleagues (7) and the JBI guidance (4, 5) refer to the process of data extraction in a scoping review as data charting*.*

§ The process of systematically examining research evidence to assess its validity, results, and relevance before using it to inform a decision. This term is used for items 12 and 19 instead of "risk of bias" (which is more applicable to systematic reviews of interventions) to include and acknowledge the various sources of evidence that may be used in a scoping review (e.g., quantitative and/or qualitative research, expert opinion, and policy document).

*From:* Tricco AC, Lillie E, Zarin W, O'Brien KK, Colquhoun H, Levac D, et al. PRISMA Extension for Scoping Reviews (PRISMAScR): Checklist and Explanation. Ann Intern Med. 2018;169:467–473. [doi: 10.7326/M18-0850.](http://annals.org/aim/fullarticle/2700389/prisma-extension-scoping-reviews-prisma-scr-checklist-explanation)

## Additional File 2: Search Strategies

**Medline Search Strategy**

| Step | Search Terms/MeSH | Search Results |
| --- | --- | --- |
| 1 | exp Dental Hygienists/ | 5894 |
| 2 | dental hygienist.mp. | 1064 |
| 3 | dental hygienists.mp. | 6623 |
| 4 | 1 or 2 or 3 | 6956 |
| 5 | workforce.mp. | 107976 |
| 6 | exp Workforce/ | 80686 |
| 7 | exp Health Workforce/ | 14413 |
| 8 | demand.mp. | 205156 |
| 9 | exp “Health Services Needs and Demand”/ | 62415 |
| 10 | supply.mp. | 614998 |
| 11 | capacity.mp. | 674630 |
| 12 | exp Capacity Building/ | 3378 |
| 13 | exp Work Capacity Evaluation/ | 6287 |
| 14 | access.mp. | 426210 |
| 15 | exp Health Services Accessibility/ | 134130 |
| 16 | availability.mp. | 307012 |
| 17 | 5 or 6 or 7 or 8 or 9 or 10 or 11 or 12 or 13 or 14 or 15 or 16 | 2246992 |
| 18 | 4 and 17 | 1077 |
| 19 | limit 18 to yr=“2013-Current” | 316 |
| 20 | limit 19 to english language | 316 |

**CINAHL Search Strategy**

| Step | Search Terms/Major Headings | Search Results |
| --- | --- | --- |
| S1 | (MH “Dental Hygienists”) | 7375 |
| S2 | “dental hygienist” | 1864 |
| S3 | “dental hygienists” | 9202 |
| S4 | S1 or S2 or S3 | 9814 |
| S5 | (MH “Workforce”) | 13905 |
| S6 | “workforce” | 35555 |
| S7 | “demand” | 59605 |
| S8 | (MH “Health Services Needs and Demand”) | 27818 |
| S9 | “supply” | 95436 |
| S10 | “capacity” | 90149 |
| S11 | (MH Work Capacity Evaluation”) | 1756 |
| S12 | “access” | 182058 |
| S13 | (MH “ Health Services Accessibility”) | 102337 |
| S14 | “availability” | 55540 |
| S15 | S5 or S6 or S7 or S8 or S9 or S10 or S11 or S12 or S13 or S14 | 526976 |
| S16 | S4 and S15 | 1042 |
| S17 | S4 and S15 (Limiters – Publication Date: 20130101-2023-12-31) | 543 |
| S18 | S4 and S15 (Narrow by Language: English) | 543 |

**Google Scholar Search Strategy**

| Search Date | Search Strategy | Search Results |
| --- | --- | --- |
| 11 May 2023 | workforce dental hygiene OR hygienist OR hygienists | 199 |
| 11 May 2023 | demand supply dental hygiene OR hygienist OR hygienists | 96 |
| 11 May 2023 | capacity dental hygiene OR hygienist OR hygienists | 39 |
| 11 May 2023 | access dental hygiene OR hygienist OR hygienists | 197 |
| 11 May 2023 | availability dental hygiene OR hygienist OR hygienists | 38 |

**Google Search Strategy**

| Search Date | Search Strategy | Search Results |
| --- | --- | --- |
| 12 May 2023 | workforce dental hygiene OR hygienist OR hygienists | 149 |
| 12 May 2023 | demand supply dental hygiene OR hygienist OR hygienists | 160 |
| 12 May 2023 | capacity dental hygiene OR hygienist OR hygienists | 165 |
| 12 May 2023 | access dental hygiene OR hygienist OR hygienists | 211 |
| 12 May 2023 | availability dental hygiene OR hygienist OR hygienists | 162 |

## Additional File 3: List of Included Articles

CL-067. Helene M. Burns, MSDH, RDH; Susan L. Tolle, MSDH, RDH; Emily A. Ludwig, MSDH, RDH; Jessica R. Suedbeck, MSDH, RDH. (2022). Attitudes of Virginia Dental Hygienists Toward Dental Therapists. Journal of Dental Hygiene, 96(1):55-63.

CL-088. Tobias E. Rodriguez, Audrey L. Galka, Ernestine S. Lacy, August D. Pellegrini, Domenica G. Sweier, Laura M. Romito. (2013). Can Midlevel Dental Providers Be a Benefit to the American Public? Journal of Health Care for the Poor and Underserved, 24(2):892-906.

CL-096. JoAnn R. Gurenlian, RDH, MS, PhD, AFAAOM; Rachel Morrissey, MA; Cameron G. Estrich, MPH, PhD; Ann Battrell, MSDH; Sue K. Bessner; Ann Lynch; Matthew Mikkelsen, MA; Marcelo W. B. Araujo, DDS, MS, PhD; Marko Vujicic, PhD. (2021). Employment Patterns of Dental Hygienists in the United States During the COVID-19 Pandemic. Journal of Dental Hygiene, 95(1):17-24.

CL-098. Chen D, Hayes MJ, Holdern ACL. (2022). Investigation into the enablers and barriers of career satisfaction among Australian oral health therapists. Community Dentistry and Oral Epidemiology, 51:301-310.

CL-104. Jean G, Kruger E, Tennant M. (2019). The distribution of allied dental practitioners in Australia: socio-economics and rurality as a driver of better health service accessibility. Australian Dental Journal, 64:153-160.

CL-128. Ayako Okada, Yuki Ohara, Yuko Yamamoto, Yoshiaki Nomura, Noriyasu Hosoya, Nobuhiro Hanada, Noriko Takei. (2021). Impact of Working Environment on Job Satisfaction: Findings from a Survey of Japanese Dental Hygienists. International Journal of Environmental Research and Public Health.

CL-165. Mackey JT. (2019). Advocacy victories across the country. ADHA Access. American Dental Hygienists Association.

CL-219. Harris JA, Simon L. (2022). Which factors are associated with the number of dental providers employed in correctional settings? Journal of Public Health Dentistry, 82:118-122.

CL-276. Monaghan NP, Morgan MZ. (2015). What proportion of dental care in care homes could be met by direct access to dental therapists or dental hygienists? British Dental Journal, 219(11):531-534.

CL-304. Langelier M, Continelli T, Moore J, Baker B, Surdu S. (2016). Expanded Scopes Of Practice For Dental Hygienists Associated With Improved Oral Health Outcomes For Adults. Health Affairs, 35(12):2207-2215.

CL-316. Draper CK. (2021). Defining the Profession in the 21st Century. Journal of Dental Hygiene, 95(6):4-5.

CL-347. Bo Rolander, Charlotte Wåhlin, Venerina Johnston, Petra Wagman & Ulrika Lindmark. (2016). Changes in division of labour and tasks within public dentistry: relationship to employees work demands, health and work ability. Acta Odontologica Scandinavica, 74(6):471-479.

CL-349. Yamalik N, Ensaldo-Carrasco E, Cavalle E, Kell K. (2014). Oral health workforce planning part 2: figures, determinants and trends in a sample of World Dental Federation member countries. International Dental Journal, 64:117-126.

CL-353. Deborah M. Lyle; Ashley Grill; Jodi Olmsted; Marilynn Rothen. (2016). National dental hygiene research agenda. Journal of Dental Hygiene, 90(Suppl 1): 43-50).

CL-364. Bell KP, Coplen AE. (2016). Evaluating the Impact of Expanded Practice Dental Hygienists in Oregon: An Outcomes Assessment. Journal of Dental Hygiene, 90(Suppl 1): 33-41.

CL-371. D.N. Teusner, N. Amarasena, J. Satur, S. Chrisopoulos and D.S. Brennan. (2016). Dental service provision by oral health therapists, dental hygienists and dental therapists in Australia: implications for workforce modelling. Community Dental Health, 33:15-22.

CL-394. Westphal Theile C. (2017). Strengths and Weaknesses of the Current Dental Hygiene Educational System. Journal of Dental Education, 81(9 Suppl):eS38-eS44.

CL-438. Charbonneau, C., J.; Kelly, D., M.; Donnelly, L., R. (2014). Exploring the views of and challenges experienced by dental hygienists practising in a multicultural society: A pilot study. Canadian Journal of Dental Hygiene, 48(4):139-146.

CL-447. Rainchuso L, Salisbury H. (2017). Public Health Dental Hygienists in Massachusetts: A Qualitative Study. Journal of Dental Hygiene, 91(3):31-36.

CL-462. Ann Battrell, MSDH, Ann Lynch, Pam Steinbach, RN, MS, Sue Bessner, Josh Snyder, and Jean Majeski. (2014). Advancing Education in Dental Hygiene. Journal of Evidence-Based Dental Practice, 14(Suppl 1): 209-221.

CL-463. Cynthia C. Gadbury-Amyot. (2014). Technology is a Critical Game Changer to the Practice of Dental Hygiene. Journal of Evidence-Based Dental Practice, 14S:240-245.

CL-471. Fried JL, Maxey HL, Battani K, Gurenlian JR, Byrd TO, Brunick A. (2017). Preparing the Future Dental Hygiene Workforce: Knowledge, Skills, and Reform. Journal of Dental Education, 81(9 Suppl):eS45-eS52.

CL-477. Anonymous. (2014). A profile of the RDH. RDH Magazine. [https://www.rdhmag.com/career-profession/ article/16404257/a-profile-of-the-rdh](https://www.rdhmag.com/career-profession/article/16404257/a-profile-of-the-rdh).

CL-486. Geisinger ML, Dershewitz SL. (2022). Worried Sick: Anxiety, depression, and the impact on dental health care workers. Journal of Dental Hygiene, 96(4):6-8.

CL-521. Carolann P. Yakiwchuk. (2013). A multi strategy approach for RDHs to champion change in long term care. Canadian Journal of Dental Hygiene, 47(2):84-88.

G-001. National Governors Association. (2013). The role of dental hygienists in providing access to oral health care. National Governors Association. https://www.nga.org/wp-content/uploads/2019/08/1401DentalHealthCare.pdf.

G-002. ADEA Advocacy and Government Relations Portfolio. (2014). Alternative workforce models. ADEA Advocacy and Government Relations Portfolio. <https://www.adea.org/uploadedFiles/ADEA/Content_Conversion_Final/policy_advocacy/Documents/emailDist/Jan_2014_Alt_Workforce_Chart.pdf>.

G-003. University of Hong Kong. (2014). Mind the gap? Projecting demand and supply of healthcare professionals. University of Hong Kong, School of Public Health.

G-004. Executive Office of Health and Human Services. (2014). Massachusetts health professions data series: dental hygienist 2013. Commonwealth of Massachusetts, Department of Public Health. <https://www.mass.gov/doc/the-massachusetts-health-professions-data-series-dental-hygienists-0/download>.

G-006. U.S. Department of Health and Human Services, Health Resources and Services Administration, National Center for Health Workforce Analysis.(2015). National and State-Level Projections of Dentists and Dental Hygienists in the U.S., 2012-2025. Rockville, Maryland. <https://bhw.hrsa.gov/sites/default/files/bureau-health-workforce/data-research/national-state-level-projections-dentists.pdf>.

G-007. McGlaston, K (State Advocacy Manager). (2015). National and state-level projections of dentists and dental hygienists in the U.S., 2012-2025. Community Catalyst. <https://www.communitycatalyst.org/wp-content/uploads/2022/11/National-and-State-Level-Projections-Summary-1.pdf>.

G-008. American Dental Hygienists Association. (2016). Dental hygienists in a hospital setting, May_June_2016. ADHA Access. <https://pubs.royle.com/publication/?m=21156&i=300966&p=16&ver=html5>.

G-011. Oral Health Workforce Research Center, Center for Workforce Studies, School of Public Health, University at Albany - State University of New York. (2016). Variation in dental hygiene scope of practice by state. University at Albany - State University of New York. <https://oralhealthworkforce.org/wp-content/uploads/2019/01/Single-Page-Layout-Final-2019.pdf>.

G-013. Canadian Dental Hygienists Association. (2018). Healthy & respectful workplace. Canadian Dental Hygienists Association. [https://www.cdha.ca/cdha/Career_folder/Healthy___Respectful_Workplace/CDHA/Career/ Healthy_Workplace/Healthy___Respectful_Workplace.aspx](https://www.cdha.ca/cdha/Career_folder/Healthy___Respectful_Workplace/CDHA/Career/Healthy_Workplace/Healthy___Respectful_Workplace.aspx).

G-014. Moibi N, Jahnke D. (2018). Oral health workforce: trends and pipeline incentives. Minnesota Department of Health. <https://www.health.state.mn.us/data/workforce/oral/docs/2018oralhealthwkfc.pdf>.

G-015. Langelier M. (2018). The impact of changing workforce models on access to oral health care services. Oral Health Workforce Research Center, Center for Workforce Studies, School of Public Health, University at Albany - State University of New York. [https://www.oralhealthworkforce.org/wp-content/uploads/2018/10/OHWRC_ Harvard_10_2018.pdf](https://www.oralhealthworkforce.org/wp-content/uploads/2018/10/OHWRC_Harvard_10_2018.pdf).

G-016. Choi E-M, Mun S-J, Chung W-G, Noh H-J. (2019). Relationships between dental hygienists' work environment and patient safety culture. BMC Health Services Research, 19(299):1-7.

G-017. National Center for Health Workforce Analysis. (2019). Oral health workforce projections, 2017-2030: dentists and dental hygienists. US Department of Health and Human Services, Health Resources and Services Administration. <https://bhw.hrsa.gov/sites/default/files/bureau-health-workforce/data-research/oral-health-2017-2030.pdf>.

G-019. Minnesota Department of Health, Office of Rural Health and Primary Care, (2019). Dental Hygienist Fact Sheet. Minnesota Department of Health. <https://www.health.state.mn.us/data/workforce/oral/docs/2019denthygchart.pdf>.

G-020. Texas Department of State Health Services, Texas Health and Human Services. (2019). Texas supply and demand dental projections, 2018-2030. Texas Health and Human Services. <https://www.dshs.texas.gov/sites/default/files/chs/hprc/publications/DSHS_DentalProjections_092019.pdf>.

G-021. Asadoorian J, Forget EL, Grace J, Torabi M. (2019). Exploring dental hygiene decision making: A qualitative study of potential organizational explanations. Canadian Journal of Dental Hygiene, 53(1):7-22.

G-022. ADHA Illinois Dental Hygienists' Association. (2020). Public health dental hygienist direct access states and year(s) direct services were enacted. ADHA Illinois Dental Hygienists' Association. <https://idha.wildapricot.org/resources/Documents/Direct%20Access%20to%20Care%20From%20DH.pdf>.

G-023. UK General Dental Council. (2020). Guidance on direct access. UK General Dental Council. <https://www.gdc-uk.org/docs/default-source/direct-access/direct-access-guidance.pdf?sfvrsn=f45d8a83_4>.

G-025. North Carolina Oral Health Collaborative. (2020). North Carolina Oral Health Collaborative Policy Brief: A Roadmap for Increasing Oral Health Access in North Carolina. North Carolina Oral Health Collaborative. <https://drive.google.com/file/d/109TC0av7PfGuQP9K4EaMnzt_RQ5DAChK/view>.

G-027. James Y. (2021). Introduction to the health workforce in Canada: Dental assistants, hygienists and therapists. In Introduction to the Health Workforce in Canada, Bourgeault IL (ed.). Canadian Health Workforce Network. [https://www.hhr-rhs.ca/images/Intro_to_the_Health_Workforce_in_Canada_Chapters/06_Dental_Asst_ Hygienists_Therapists.pdf](https://www.hhr-rhs.ca/images/Intro_to_the_Health_Workforce_in_Canada_Chapters/06_Dental_Asst_Hygienists_Therapists.pdf)

G-028. Minnesota Department of Health, Office of Rural Health and Primary Care. (2021). Understanding collaborative dental hygiene practice in Minnesota. Minnesota Department of Health. <https://www.health.state.mn.us/data/workforce/oral/docs/2021ucdhpmn.pdf>.

G-029. Langelier M, Moore J, Continelli T. (2021). Dental hygiene scope of practice: Developing a measurement tool, finding associations with oral health outcomes, and informing policy change. Oral Health Workforce Research Center, Center for Workforce Studies, School of Public Health, University at Albany - State University of New York. <https://www.chwsny.org/wp-content/uploads/2021/06/Scope-of-Practice-Symposium_2021_Final.pdf>.

G-031. Canadian Dental Hygienists Association. (2022). Submission from the Canadian Dental Hygienists Association to the House of Commons Standing Committee on Health: RE study on Canada's health workforce. Canadian Dental Hygienists Association. <https://www.ourcommons.ca/Content/Committee/441/HESA/Brief/BR11741959/br-external/CanadianDentalHygienistsAssociation-e.pdf>.

G-032. Ghoneim A, Parbhakar KK, Farmer J, Quinonez C. (2022). Healthy and respectful workplaces: the experiences of dental hygienists in Canada. JDR Clinical and Translational Research, 7(2):194-204.

G-033. Food and Health Bureau: Hong Kong Special Administrative Region Government. (2017). Strategic review on healthcare manpower planning and professional development. Hong Kong Special Administrative Region Government. [https://www.healthbureau.gov.hk/download/press_and_publications/otherinfo/180500_sr/e_sr_ final_report.pdf](https://www.healthbureau.gov.hk/download/press_and_publications/otherinfo/180500_sr/e_sr_final_report.pdf).

G-035. Traul R. (2023). 2019-2020 workforce survey of dental hygienists. Florida Department of Health. [https://www.floridahealth.gov/programs-and-services/community-health/dental-health/reports/_documents /florida-workforce-survey-report-of-dental-hygienists-2019-2020.pdf](https://www.floridahealth.gov/programs-and-services/community-health/dental-health/reports/_documents/florida-workforce-survey-report-of-dental-hygienists-2019-2020.pdf).

G-036. Medlock C, Jamison R, Maxey HL. (2023). 2022 Indiana Dental Hygienist Brief (2023). Bowen Center for Health Workforce Research and Policy. Indiana University School of Medicine. <https://scholarworks.iupui.edu/items/f85020cc-c664-492c-976c-87aba9d9ac5b/full>.

GS-017. Isman BA, Farrell CM. (2014). Are Dental Hygienists Prepared to Work in the Changing Public Health Environment? Journal of Evidence-Based Dental Practice, 1(4S):183-190.

GS-026. Hannah L. Maxey, PhD, MPH, RDH; Christine Farrell, RDH, BSDH, MPA; Anne Gwozdek, RDH, MA. (2017). Exploring Current and Future Roles of Non-Dental Professionals: Implications for Dental Hygiene Education. Journal of Dental Education, 81(9):eS53:eS58.

GS-036. Ji-Hyoung Han, Kwui-Sook Song, and Sun-Jung Shin. (2018). Factors Affecting Job Satisfaction of Clinical Dental Hygienists. Journal of Dental Hygiene Science, 18(6):374-379.

GS-046. Richard D. Holmes, Bryan Burford and Gillian Vance. (2020). Development and retention of the dental workforce: findings from a regional workforce survey and symposium in England. BMC Health Services Research, 20(255):1-11.

GS-050. Brandon Vick. (2015). Career satisfaction of Pennsylvanian dentists and dental hygienists and their plans to leave direct patient care. Journal of Public Health Dentistry, 76:113-121.

GS-052. Diaz J, Boyd LD, Giblin-Scanlon L, Smethers R, Vineyard J. (2022). Experiences and characteristics of men working in dental hygiene. International Journal of Dental Hygiene, 20:185-192.

GS-053. Reinders JJ, Krinjnen WP, Onclin P, van der Schans CP, Stegenga B. (2017). Attitudes among dentists and dental hygienists towards extended scope and independent practice of dental hygienists. International Dental Journal, 67:46-58.

GS-055. Julie C. Reynolds, DDS, MS; Susan C. McKernan, DMD, MS, PhD; Raymond A. Kuthy, DDS, MPH. (2021). Predictors of Multiple Jobholding among Dental Hygienists in the State of Iowa. Journal of Dental Hygiene, 95(1):43-49.

GS-059. JI Virtanen; E Pellikka; S Singh; E Widström. (2016). The professional role of a dental hygienist in Finland – educators’ views. International Journal of Dental Hygiene, 14:231-238.

GS-069. Reynolds, J. C., McKernan, S. C., Adekugbe, O., Sukalski, J. M. C., & Kuthy, R. A. (2019). Dental Hygiene Workforce in Iowa: Current Capacity and Implications for Access to Care for the Underserved. University of Iowa Public Policy Center. https://doi.org/10.17077/rep.001115. <https://ppc.uiowa.edu/sites/default/files/dental_hygiene_workforce_capacity_access.pdf>.

GS-086. Badal M. Patel; Linda D. Boyd; Jared Vineyard; Lisa LaSpina. (2021). Job Satisfaction, Burnout, and Intention to Leave among Dental Hygienists in Clinical Practice. Journal of Dental Hygiene, 95(2):28-35.

GS-098. DeRosa Hays R, Moglia Willis S. (2021). The Baccalaureate as the Minimum Entry-Level Degree in Dental Hygiene. Journal of Dental Hygiene, 95(6):46-53.

GS-107. Yoshiaki Nomura, Ayako Okada, Jun Miyoshi, Masaru Mukaida, Eriko Akasaka, Keietsu Saigo, Hideki Daikoku, Hidenori Maekawa, Tamotsu Sato, Nobuhiro Hanada. (2018). Willingness to Work and the Working Environment of Japanese Dental Hygienists. International Journal of Dentistry, https://doi.org/10.1155/2018/2727193 pp.1-9.

GS-118. M. K. Ross (2022). A forgotten workforce? British Dental Journal, 233(6):440-441.

GS-150. Muroga R, Tsuruta J, Morio I. (2015). Disparity in perception of the working condition of dental hygienists between dentists and dental hygiene students in Japan. International Journal of Dental Hygiene, 13:213-221.

GS-167. Tracee S. Dahm; Ann Bruhn; Margaret LeMaster. (2015). Oral Care in the Long-Term Care of Older Patients: How Can the Dental Hygienist Meet the Need? Journal of Dental Hygiene, 89(4):229-238.

GS-180. Yoshiaki Nomura, Ayako Okada, Yuko Yamamoto, Erika Kakuta, Hiroshi Tomonari, Noriyasu Hosoya, Nobuhiro Hanada, Naomi Yoshida, Noriko Takei. (2020). Factors Behind Leaving the Job and Rejoining it by the Japanese Dental Hygienist. The Open Dentistry Journal, 14:355-361.

GS-185. Catlett A. (2016). Attitudes of Dental Hygienists towards Independent Practice and Professional Autonomy. Journal of Dental Hygiene, 90(4):249-256.

GS-190. S Kimberly Haslam; Alma Wade; Lindsay K Macdonald; Jennifer Johnson; Leigha D Rock. (2022). Burnout syndrome in Nova Scotia dental hygienists during the COVID-19 pandemic: Maslach Burnout Inventory. Canadian Journal of Dental Hygiene, 56(2):63-71.

GS-209. Kang H-S, Jung Y-R, Cho Y-Y. (2018). Survey on the working environment of national clinical dental hygienists. Journal of Korean Society of Dental Hygiene, 18(6):863-878.

GS-221. Lee H-J, Shin S-J, Bae S-M, Shin B-M. (2019). Issues and Challenges of Dental Hygienist Workforce Policy in Korea. Journal of the Korea Contents Association, 19(2):409-423.

GS-242. Laurie V. Bercasio; Dorothy J. Rowe; Alfa-Ibrahim Yansane. (2020). Factors Associated with Burnout among Dental Hygienists in California. Journal of Dental Hygiene, 94(6):40-48.

GS-248. Kim H-J, Kim Y-J, Kim M-H. (2014). A Study of Factors Related to Job Satisfaction Affecting Service Year: A Dental Hygienist in Seoul. Journal of Dental Hygiene Science, 14(4):510-515.

GS-249. Elizabeth J. Brown. (2016). Dental Hygienist Providers in Long-Term Care: Meeting the Need. Journal of Evidence-Based Dental Practice, 16S:77-83.

GS-257. Falk Schwendicke; Ralf Jäger; Wolfgang Hoffmann; Rainer A. Jordan; Neeltje van den Berg. (2016). Estimating spatially specific demand and supply of dental services: a longitudinal comparison in Northern Germany. Journal of Public Health Dentistry, 76:269-275.

GS-285. Seong M-G, Kim Y-R. (2019). Comparison of job satisfaction, turnover intention, and job performance by dental hygienists' clinical assistance and preventive work. Journal of Korean Society of Dental Hygiene, 19(2):221-229.

GS-289. Jeong-A Yang; Soon-Ryun Lim; Young-Sik Cho. (2017). Development of scale of long-term employment intention for dental hygienist. Journal of Korean Society of Dental Hygiene, 17(6):1025-1035.

GS-302. Darlene M. Jones; Sonya R. Miller. (2018). Effectiveness of an Educational Module on Dental Hygiene Students' Attitudes Towards Persons with Disabilities. Journal of Dental Hygiene, 92(4):27-34.

GS-327. Z Kanji; DM Laronde. (2018). Motivating influences and ability-based outcomes of dental hygiene baccalaureate education in Canada. International Journal of Dental Hygiene, 16:329-339.

GS-335. Naughton DK. (2014). Expanding Oral Care Opportunities: Direct Access Care Provided by Dental Hygienists in the United States. Journal of Evidence-Based Dental Practice, 1(4S):171-182.

GS-352. Coplen AE, Bell KP. (2015). Barriers Faced by Expanded Practice Dental Hygienists in Oregon. Journal of Dental Hygiene, 89(2):91-100.

GS-384. Jun-Yeong Kwon and Su-Young Lee. (2016). Relationship of between Task Performance, Job Satisfaction, and Organizational Contribution of Dental Hygienists. Journal of Dental Hygiene Science, 16(4):302-309.

GS-394. Lee S-S. (2013). Professionalism and job satisfaction in dental hygienists. Journal of Korean Society of Dental Hygiene, 13(4):535-542.

GS-403. Chen J, Meyerhoefer CD, Timmons EJ. (2020). The Effects of Dental Hygienist Autonomy on Dental Care Utilization. Center for Growth and Opportunity, Utah State University. <https://www.thecgo.org/research/the-effects-of-dental-hygienist-autonomy-on-dental-care-utilization>.

GS-413. Dennis Chen; Melanie Hayes; and Alexander Holden. (2021). A global review of the education and career pathways of dental therapists, dental hygienists and oral health therapists. British Dental Journal, https://doi.org/10.1038/s41415-021-2836-z pp533-538.

GS-438. Mi-Hae Yun; Hee-Hong Min. (2019). Factors influencing burnout in clinical dental hygienists. Journal of Korean Society of Dental Hygiene, 19(6):975-982.

GS-445. Muneeb A. Shaikh; Marita R. Inglehart. (2018). Dental and Dental Hygiene Students’ Career Choice Motivations in 2009–17: A Mixed Methods Approach. Journal of Dental Education, 82(8):848-856.

GS-467. Kathryn Bell. (2019). Interprofessional Education: Preparing dental hygienists to practice in the evolving health care world. Journal of Dental Hygiene, 93(5):4-5.

GS-477. Min H-H, Jeon J-H, Kim Y-S. (2015). Influencing factors of turnover intention in the clinical dental hygienists. Journal of Korean Society of Dental Hygiene, 15(5):831-839.

GS-482. Kim J-H, Hwang T-Y. (2013). Development of job performance assessment tool for dental hygienists. Journal of Korean Society of Dental Hygiene, 13(5):713-725.

GS-504. Park J-H, Lim S-R. (2020). A Qualitative Study on Job Satisfaction of Dental Hygienists with Low Experience. Journal of Dental Hygiene Science, 20(3):163-170.

GS-506. Lee D-S, Han G-S. (2018). Problems to Solve and Job Enlargement on the Inclusion of Dental Hygienists in the Category of Medical Personnel. Journal of Dental Hygiene Science, 18(6):340-348.

GS-508. Susanne Sunell; Denise M Laronde; Zul Kanji (2021). Fourth-year dental hygiene students’ educational preparedness: Self-confidence ratings of the Canadian Dental Hygienists Association baccalaureate competencies (2017-2019). Journal of Dental Education, 85:768-777.

GS-522. Department of Dental Hygiene, Daegu Health College; Department of Dental Hygiene, Kyungwoon University. (2013). Perceptions of dental hygienists toward digital dentistry. Journal of Korean Society of Dental Hygiene, 13(6):909-916.

GS-737. Joshua D. D. Quach; Latha S. Davda; David R. Radford; and Chris Louca. (2020). Are dental schools doing enough to prepare dental hygiene & therapy students for direct access? British Dental Journal, https://doi.org/10.1038/s41407-020-0471-1: pp36-37.

ML-011. Ishiguro-Matsumoto, A, Stegaroiu, R, Suwama, K, Shibata, S, Yoshihara, A, Ohuchi, A. (2023). Career outcomes and satisfaction among graduates of a 4-year oral health and welfare baccalaureate program at Niigata University, Japan: A cross-sectional study. International Journal of Dental Hygiene, 00:1-12.

ML-012. Hallett G; Witton R; Mills I. (2022). A survey of mental wellbeing and stress among dental therapists and hygienists in South West England. British Dental Journal, https://doi.org/10.1038/s41415-022-5357-5 pp1-6.

ML-013. Jackson-Collins C, Boyd LD, Jenkins SJ. (2022). Clinical Dental Hygienists' Experience Returning to Work After Closure of Dental Offices Due to COVID-19: A qualitative study. Journal of Dental Hygiene, 96(6):6-14.

ML-025. Rachel W. Morrissey, MA; JoAnn R. Gurenlian, RDH, MS, PhD, AFAAOM; Cameron G. Estrich, MPH, PhD; Laura A. Eldridge, MS; Ann Battrell, MSDH; Ann Lynch; Matthew Mikkelsen, MA; Brittany Harrison, MA; Marcelo W. B. Araujo, DDS, MS, PhD; Marko Vujicic, PhD. (2022). Employment Patterns of Dental Hygienists in the United States During the COVID-19 Pandemic: An update. Journal of Dental Hygiene, 96(1):27-34.

ML-031. Johnson K, Gurenlian J, Garland K, Freudenthal J. (2020). State Licensing Board Requirements for Entry into the Dental Hygiene Profession. Journal of Dental Hygiene, 94(2):54-65.

ML-032. Lawlor S. (2013). Interprofessional practice: Enhancing the dental hygienist's role. Canadian Journal of Dental Hygiene, 47(1):11-13.

ML-042. Antonio Javier Expósito-Delgado, Verónica Ausina-Márquez, María Victoria Mateos-Moreno, Elena Martínez-Sanz, María del Carmen Trullols-Casas, María Eulalia Llamas-Ortuño, José María Blanco-González, Teresa Almerich-Torres, Manuel Bravo and Yolanda Martínez-Beneyto . (2022). Delivery of Health Care by Spanish Dental Hygienists in Private and Public Dental Services during the COVID-19 De-Escalation Phase (June 2020): A Cross-Sectional Study. Specialty Care Dentistry, 42:592-598.

ML-058. Yuko Yamamoto, Yoshiaki Nomura, Ayako Okada, Erika Kakuta , Naomi Yoshida , Noriyasu Hosoya, Nobuhiro Hanada and Noriko Takei. (2021). Improvement of Workplace Environment That Affects Motivation of Japanese Dental Hygienists. International Journal of Environmental Research and Public Health, 18(1309):1-12.

ML-073. Olson H, Ratnayake J, Veerasamy, Quaranta A, Meldrum A. (2020). Working characteristics for practising dental hygienists in two countries in different hemispheres. International Journal of Dental Hygiene, 20:209-218.

ML-077. Vy Nguyen, DDS, MPH1 ; Marlon Daniel, MPH, MHA2; Renée Joskow, DDS, MPH3; Connie Lu, MPH4; Xiao Chen, PhD4; Weihao Zhou, MS4; Sue Lin, MS, PhD1; Alek Sripipatana, PhD, MPH1, Suma Nair, MS, PhD, RD1; Nadereh Pourat, PhD4. (2020). Impact of oral health service expansion funding at health centers in the United States. Journal of Public Health Dentistry, 80:304-312.

ML-125. Battani K. (2018). What does research tell us about the future of dental hygiene? Journal of Dental Hygiene, 92(2):4-5.

ML-128. LaSpina L, August J, Morrison P, Soal K, Pelullo K. (2017). A Survey of Massachusetts Dental Hygienists: Practice Settings, Interest in Educational Advancement, and Career Satisfaction in All Settings. Journal of Dental Hygiene, 91(2):62.

ML-134. Biazar J. (2020). How we practice today: direct access. ADHA Access. American Dental Hygienists Association.

ML-165. Maxey HL, Norwood CW, O'Connell JB, Liu Z. (2017). Impact of State Workforce Policies on Underserved Patients' Access to Dental Care: A longitudinal study. Journal of Dental Hygiene, 91(5):26-39.

ML-168. Isong I, Dantas L, Gerard M, Kuhlthau K. (2014). Oral Health Disparities and Unmet Dental Needs among Preschool Children in Chelsea, MA: Exploring Mechanisms, Defining Solutions. Journal of Oral Hygiene Health, 2:1-18.

ML-173. Simmer-Beck M, Walker M, Gadbury-Amyot C, Liu Y, Kelly P, Branson B. (2015). Effectiveness of an Alternative Dental Workforce Model on the Oral Health of Low-Income Children in a School-Based Setting. American Journal of Public Health, 105(9):1763-1769.

ML-219. Battrell A, Lynch A, Steinbach P. (2016). The American Dental Hygienists' Association Leads the Profession into 21st Century Workforce Opportunities. Journal of Evidence-Based Dental Practice, 1(6S):4-10).

ML-242. Teusner DN, Amarasena N, Satur J, Chrisopoulos S, Brennan DS. (2015). Applied scope of practice of oral health therapists, dental hygienists and dental therapists. Australian Dental Journal, 61:342-349.

ML-245. Y Usui, H Miura. (2015). Workforce re-entry for Japanese unemployed dental hygienists. International Journal of Dental Hygiene, 13:74-78.

ML-256. Feng X, Sambamoorthi U, Wiener RC. (2017). Dental workforce availability and dental services utilization in Appalachia: a geospatial analysis. Community Dentistry and Oral Epidemiology, 45:145-152.

ML-285. Lindquist L, Seleskog B, Wardh I, von Bultzingslwen I. (2013). Oral care perspectives of professionals in nursing homes for the elderly. International Journal of Dental Hygiene, 11:298-305.

ML-315. Amarasena N, Teusner DN, Brennan DS, Satur J. (2018). Practice characteristics and service provision rates of dental hygienists in Australia. International Journal of Dental Hygiene, 16:125-133.

TS-001. Stormon N, Tran C & Suen B. (2021). Australian Oral Health Workforce: The Oral Health Professions Workforce Survey 2020. Brisbane: The University of Queensland. The Australian Dental and Oral Health Therapists Association Ltd. And The Dental Hygienists Association of Australia Ltd. <https://espace.library.uq.edu.au/view/UQ:da128e4>.

TS-004. Canadian Institute for Health Information. (2022). Health Workforce in Canada, 2017 to 2021: Overview — Data Tables. Ottawa, ON: CIHI. <https://www.cihi.ca/sites/default/files/document/health-workforce-canada-2017-2021-overview-data-tables-en.xlsx>.

TS-006. Ontario Dental Association. (2023). Remote Areas Program. Ontario Dental Association. <https://www.oda.ca/about-us/remote-areas-program>.

TS-007. Ordre des Hygiéists Dentaires du Québec. (2022). Rapport annuel 2021-2022 - Ordre des hygienistes dentaires du Quebec. Ordre des Hygiéists Dentaires du Québec (OHDQ). <https://ohdq.com/wp-content/uploads/2022/10/Rapport_annuel_2021-2022_Publie-1.pdf>.

TS-010. Crozier S. (2023). Recruiting hygienists, assistants still challenging for practice owners. American Dental Political Action Committee. <https://adanews.ada.org/ada-news/2023/july/recruiting-hygienists-assistants-still-challenging-for-practice-owners/>.

TS-011. International Federation of Dental Hygienists. (2020). Working abroad as a dental hygienist: Australia. <https://ifdh.org/australia>.

TS-012. International Federation of Dental Hygienists. (2020). Working abroad as a dental hygienist: Japan. <https://ifdh.org/japan>.

TS-013. International Federation of Dental Hygienists. (2023). Working abroad as a dental hygienist: Canada. <https://ifdh.org/canada>.

TS-014. International Federation of Dental Hygienists. (2013). Working abroad as a dental hygienist: Korea. <https://ifdh.org/korea>.

TS-015. International Federation of Dental Hygienists. (2014). Working abroad as a dental hygienist: Switzerland. <https://ifdh.org/switzerland>.

TS-016. International Federation of Dental Hygienists. (2022). Working abroad as a dental hygienist: UK. <https://ifdh.org/united-kingdom>.

TS-017. International Federation of Dental Hygienists. (2023). Working abroad as a dental hygienist: USA. <https://ifdh.org/usa>.

TS-021. American Dental Hygienists Association. (2023). License portability. American Dental Hygienists Association. <https://www.adha.org/advocacy/license-portability/>.

TS-024. Dental Board, Australian Health Practitioner Regulation Agency. (2023). Dental Board of Australia Registrant data: Reporting Period: 01 January 2023 to 31 March 2023. Australian Health Practitioner Regulation Agency. <https://www.dentalboard.gov.au/About-the-Board.aspx>.

TS-025. Brocklehurst P. (2022). How do we meet the future dental public health challenges before us?. International Symposium on Dental Hygiene 2022. <https://isdh2022.com/programme-schedule>.

TS-026. Grandjean M-L. (2022). The future of the geriatric oral health care is in the hands of the hygienists. International Symposium on Dental Hygiene 2022. <https://isdh2022.com/programme-schedule>.

TS-027. Moravec L, Betts K. (2022). Integrating oral health care into primary care well child visits: An interprofessional model of care. International Symposium on Dental Hygiene 2022. <https://isdh2022.com/programme-schedule>.

TS-030. Young K, Knight D, Atkinson J, Lewis M. (2022). Exploring alternative workforce models with dental hygiene students (Session O-107). International Symposium on Dental Hygiene 2022. <https://isdh2022.com/programme-schedule>.

TS-031. Neville P. (2022). The academization of dental hygiene: exploring the experiences of dental hygiene educators and leaders overseeing the transition to a degree programme. International Symposium on Dental Hygiene 2022. https://isdh2022.com/programme-schedule.

TS-032. Boge E. (2022). Benefits of a dental hygienist obtaining a baccalaureate degree: A qualitative research study of licensees in five midwestern states (Session O-116). International Symposium on Dental Hygiene 2022. <https://isdh2022.com/programme-schedule>.

TS-039. Anishchuk S. (2022). Examining the relationship between burnout and empathy in healthcare professionals: A systematic review (Session O-312). International Symposium on Dental Hygiene 2022. <https://isdh2022.com/programme-schedule>.

TS-040. Spring C, Larsson L. (2022). Interprofessional mobile outreach to improve health, community involvement and support. International Symposium on Dental Hygiene 2022. <https://isdh2022.com/programme-schedule>.

TS-042. International Federation of Dental Hygienists. (2020). Working abroad as a dental hygienist: Finland. <https://ifdh.org/finland>.

TS-043. International Federation of Dental Hygienists. (2020). Working abroad as a dental hygienist: Sweden. <https://ifdh.org/sweden>.

TS-044. International Federation of Dental Hygienists. (2017). Working abroad as a dental hygienist: Germany. <https://ifdh.org/germany>.

TS-046. International Federation of Dental Hygienists. (2023). Working abroad as a dental hygienist: Spain. <https://ifdh.org/spain>.

X-001. Centre for Workforce Intelligence. (2014). Securing the future workforce supply: Dental care professionals stocktake. London, UK: Centre for Workforce Intelligence. <https://assets.publishing.service.gov.uk/media/5a818686ed915d74e33feaf2/CfWI_Dental_care_professionals_stocktake.pdf>

X-002. Dental Workforce Advisory Group for England. (2019). The future oral and dental workforce for England: Liberating human resources to serve the population across the life-course. London, UK: Health Education England. <https://www.hee.nhs.uk/sites/default/files/documents/FDWF%20Report%20-%207th%20March%202019.pdf>

X-003. Kanji Z, Laronde DM. (2018). Career outcomes of dental hygiene baccalaureate education: a study of graduates' professional opportunities, further education, and job satisfaction. Journal of Dental Education, 82(8): 809-818.
